# Ankle Magnetic Resonance Imaging in Juvenile Idiopathic Arthritis Versus Non-Juvenile Idiopathic Arthritis Patients with Arthralgia

**DOI:** 10.3390/jcm11030760

**Published:** 2022-01-30

**Authors:** Monika Ostrowska, Emil Michalski, Piotr Gietka, Małgorzata Mańczak, Magdalena Posadzy, Iwona Sudoł-Szopińska

**Affiliations:** 1 Department of Radiology, National Institute of Geriatrics, Rheumatology and Rehabilitation, 02-637 Warsaw, Poland; monique.ostrowska@gmail.com (M.O.); sudolszopinska@gmail.com (I.S.-S.); 2 Clinic of Pediatric Rheumatology, National Institute of Geriatrics, Rheumatology and Rehabilitation, 02-637 Warsaw, Poland; malgieta1@gmail.com; 3 Department of Gerontology, Public Health and Didactics, National Institute of Geriatrics, Rheumatology and Rehabilitation, 02-637 Warsaw, Poland; m.manczak@op.pl; 4 Indywidualna Praktyka Lekarska, Magdalena Posadzy, 61-426 Poznań, Poland; magdalenaposadzy@gmail.com

**Keywords:** juvenile idiopathic arthritis, ankle arthritis, magnetic resonance imaging, scoring

## Abstract

This retrospective case–control study aimed to evaluate whether Magnetic Resonance Imaging (MRI) enables differentiation of ankle arthritis in Juvenile Idiopathic Afrthritis JIA from ankle arthralgia of unknown aetiology in patients clinically suspected of JIA. Forty-four children, at ages 5–16, who underwent MRI of the ankle from January 2016 to March 2021 for clinically suspected active ankle arthritis in the course of JIA were included. MRI findings in both groups—patients with the final diagnosis of JIA and without final diagnosis of JIA—were compared and scored. The sum of the scores of 22 ankle lesions in an individual patient (active, destructive and developmental), so-called the MRI summarized score, was calculated and tested in terms of the most optimal diagnosis of JIA. Interobserver agreement was calculated. Inflammatory features were seen on MRI in 38 out of all the included patients (86%). The most common lesions in both groups were effusion in the tibio-talar joint (68% in JIA and 64% in the arthralgia group) and effusion in subtalar joint (64% in JIA vs. 59% in the arthralgia group). In general, more lesions were identified in the JIA group than in non-JIA. However, only tenosynovitis was significantly more common in the JIA vs. non-JIA group (*p* = 0.031). The MRI summarized score did not allow for discrimination between ankle arthritis in JIA from non-JIA patients; the best levels of sensitivity (32%), specificity (91%), positive predictive value PPV (78%) and negative predictive value NPV (57%) were achieved only at the cut-off point of 10.

## 1. Introduction

Juvenile idiopathic arthritis (JIA) is a heterogenous group of chronic nonpyogenic inflammatory arthritides with persistent symptoms for at least 6 weeks that are present before the child is 16 years old [1,2,3,4]. It is the most common form of childhood arthritis [5,6,7]. In certain JIA subtypes, the disease is rapidly progressive, and if diagnosed with a delay and left untreated, may lead to structural damage and permanent impairment of physical function [8].

Joint damage in JIA results from synovitis with formation of pannus, which, in the so-called outside-in mechanism, destroys cartilage and bone, leading to subchondral erosions, inflammatory cysts, and ankylosis [6]. A similar inflammatory process, osteitis, starts within the subchondral bone marrow and, through the inside-out mechanism, first leads to subchondral cysts formation, with further evolution to erosions and hyaline cartilage damage [9,10,11]. Another location of disease, typical for the juvenile enthesitis-related arthritis (ERA) subtype of JIA, are tendons, ligaments, and capsule entheses with inflammation (enthesitis) may lead to bone reaction with the formation of cysts and erosions and peri-entheseal soft tissue involvement [12]. Active inflammation may also concern tendons’ sheaths; tenosynovitis may lead to secondary tendinitis with possible tendon tears. Lastly, inflammation of the intra- or extraarticular fat tissue is recognized as another important pathogenetic factor in rheumatology [13]. Early detection of all disease locations is important to assess the advancement of disease with disease progression and to adapt appropriate treatment [14].

After the knee, the ankle and foot are the joints most commonly affected by JIA [4,15]. Clinical symptoms most frequently include arthralgia, and/or joint edema, and/or tenderness. Differentiation between JIA-related and non-JIA pathologies remains challenging, since there are no specific clinical or laboratory findings enabling differentiation between JIA and traumatic, overused lesions, or septic, reactive, post-infectious, or even malignant lesions affecting the ankle or foot [16].

Magnetic Resonance Imaging (MRI) is considered to be the most sensitive imaging tool for the detection of synovitis as well as for the detection of cartilage lesions, bone erosions, and bone marrow pathologies [17,18,19,20,21]. Despite high sensitivity, the specificity of MRI findings remains problematic, since a number of other pathologies follow MRI patterns similar to JIA. The most common pathologies in JIA are thought to be effusions and synovitis. Most accurately joint effusions and synovitis are distinguished on postcontrast MRI images using gadolinium injection [1,22]. Since there is no evidence to date that gadolinium accumulation in the brain and kidneys is harmful to the human body [23,24], with hypothetical cumulative and long term effects of retained gadolinium, it warrants special attention while making decisions when performing contrast examination in children and young adults [25], and indicates the need to investigate the non-contrast MRI to assess inflammation in the pediatric population [23,24].

As it is often difficult to differentiate fluid and synovitis by MRI without intravenous administration of contrast, the term “effusion/synovial thickening” was introduced where these two inflammatory features are considered all together as a single item [25]. Other active inflammatory lesions that may be seen in children with JIA include osteitis, bursitis, enthesitis, myositis, and panniculitis. Destructive inflammatory lesions developing in the course of JIA include cysts, erosions, joint space narrowing, chondromalacia, ankylosis, and secondary osteoarthritis. Joint inflammation overlapping natural bone growth in juveniles may lead to growth disturbances and developmental disorders [9,10,15].

So far, there are only single papers describing active and destructive inflammatory lesions in the ankle in JIA, and they focus on primary findings [8,26]. This study aimed to evaluate the full spectrum of possible JIA-related pathologies in MRI, including active, destructive, and developmental lesions, and to test if MRI allows ankle arthritis in JIA to be differentiated from ankle arthralgia of unknown aetiology. It was hypothesized that non-contrast MRI of the ankle can differentiate between these two groups of patients.

## 2. Patients and Methods

### 2.1. Patients

This retrospective single-center study included 44 children aged 5–16 with clinically suspected active ankle arthritis who underwent MRI of the ankle from January 2016 to March 2021. For patients who underwent multiple ankle MRIs, only the first exam was included. Both MRI and clinical evaluations were performed in the authors’ referential center for pediatric rheumatology.

On clinical evaluation children presented with ankle pain, swelling, tenderness, and/or limitation of movement associated with pain persisting more than 6 weeks [27].

Children with diagnoses other than inflammatory arthritis (e.g., tumour, trauma, septic arthritis, osteomyelitis, including chronic recurrent multifocal osteomyelitis), as well as children with the history of intraarticular corticosteroid injection or radionuclide synovectomy performed within the last six months were excluded.

The clinical data were collected, including demographic information concerning age and sex and the final diagnosis.

Parents or legal guardians of all patients gave informed consent to take part in the study. The study was performed in accordance with the Declaration of Helsinki and was approved by the local ethics committee (KBT-3/5/2018).

### 2.2. MRI Protocol and Interpretation of Imaging Features

MRI of the ankle was performed on a 1.5 Tesla system (Siemens Avanto) in a dedicated 8 channels coil. Patients were examined in a supine position. No sedation was used. Intravenous contrast agent was not used. The following sequences and planes were applied: T2-weighted (w), PD (Proton Density) and PD-w with fat saturation (FS) in axial plane, PD FS and T1-w in sagittal plane, and T2 TIRM (Turbo Inversion Recovery Magnitude) and PD-w in coronal plane. Sequences, planes of imaging, slice thickness, and the remaining technical information regarding the MRI examination are shown in Table 1.

The range of examination in all sequences covered the area from the tibiotalar joint to the metatarso-phalangeal (MTP) joints.

The following joints were covered: tibiotalar, subtalar, talonavicular, calcaneocuboid, “cuneocuboid joint”, naviculocuneiform (3 articulations), tarsometatarsal (Lisfranc joint; 5 articulations), and MTP joints. Bone marrow edema was assessed in the distal end of tibia and fibula, in talus, calcaneus, naviculare, medial, intermedium, and the lateral cuneiforms, cuboideum, proximal ends of the metatarsal bones 1–5.

The evaluated tendons included: tibialis anterior, extensor digitorum longus, extensor hallucis longus, tibialis posterior, flexor hallucis longus, flexor digitorum longus, peroneus longus, and peroneus brevis.

Out of all distal entheses, the entheses of the long extensors and long flexors of toes and halluces, and the forefoot in general, were not evaluated according to the study protocol for the ankle joint.

Entheses of the interosseous ligaments at the level of the ankle and midfoot and hindfoot entheses of the Achilles tendon and plantar fascia were included.

Two hindfoot bursae of the Achilles tendon and subcutaneous bursa of the heel were assessed.

Images were evaluated with the aim of identifying and scoring active, destructive, inflammatory lesions, as well as developmental lesions of the ankle and midfoot joint, listed in Table 2. MRI definitions for active and chronic inflammatory lesions were based on the Panwar et al. [25] and on the Arthritis Subcommittee of the European Society of Musculoskeletal Radiology (ESSR) recommendations [28].

The term “effusion/synovial thickening” was introduced by Panwar et al. [25], defined as “an increased amount (greater than physiologic) of high signal intensity within the joint space distending the joint capsule on fluid sensitive sequences”. Bone marrow edema (BME) is of high signal on T2-w and PD-w images, and is best visualized by T2 FS or STIR/TIRM sequences, hypointense on T1w images [28]. Enthesis is hyperintense on T2 and PDw images, best visualized by T2 FS or STIR/TIRM sequences, and is hypointense on T1w images. The bony part of an enthesis may show BME [28]. Bone erosions are sharply marginated trabecular bone defects with disrupted cortical bone continuity, seen in at least two planes, with low signal intensity on T1-w images. When active (filled with active synovitis) they are of low signal on T1-w and high signal intensity on fluid sensitive sequences. Intraosseous cysts present as high signal intensity foci on T2-w images and low signal intensity on T1-w images, and they are better delineated compared with ill-defined areas of BME [28].

Remaining destructive lesions included: joint space narrowing, ankylosis, osteophytes, sclerotization, and avascular necrosis (AVN).

Developmental disorders included: bone remodeling, premature closure of physis, coalition, accessory bone, and Stieda process.

The images were independently evaluated and scored by two radiologists, both with 15 years of experience in MSK imaging (10 years in the rheumatological center), blinded to clinical and laboratory data.

All lesions in each ankle were scored in a binary way (0–1) except for effusions/synovial thickening and BME (scores 0–3) and tenosynovitis (scores 0–2). Final MRI diagnosis and the scorings for JIA-confirmed patients vs. patients without final diagnosis of JIA were established as a consensus. Results are included in Table 3.

In addition, the MRI summarized score was calculated as the sum of scores of all 22 ankle and foot lesions in an individual patient to test which value provides the most optimal diagnosis in terms of high sensitivity, specificity, and positive and negative predictive values. The results are presented in Table 4.

### 2.3. Statistical Analysis

The MRI lesions are presented as numbers and percentages. The chi-squared test and a chi-squared test with Yates’ correction were used. The receiver operating characteristic (ROC) curve analysis was used to verify the discriminant ability of the MRI summarized score. The sensitivity, specificity, positive predictive value (PPV), and negative predictive value (NPV) of the created MRI score were calculated. The level of statistical significance was set at *p*  <  0.05. Statistical analyses were performed with Statistica v. 13.1 (Dell Inc. 2016, Tulsa, OK, USA). Interobserver agreement was calculated using Cohen’s kappa coefficient. Kappa values below 0.20 were considered poor agreement, 0.21–0.40 fair, 0.41–0.60 moderate, 0.61–0.80 good, and 0.81–1.00 very good [29]. The final score was established by consensus.

## 3. Results

This study included 44 children with clinically suspected ankle arthritis in whom MRI of ankles and feet were performed between January 2017 and March 2021. The mean age of patients was 12.7 (range: 5–17) with female predominance (35 girls and 9 boys).

Out of 44 included patients, JIA was confirmed in 22 patients (50%) including 14 with oligoarthritis, 6 with undifferentiated JIA, 1 patient with systemic JIA, and 1 patient with the ERA subtype. In the remaining 22 (50%) patients, JIA was excluded.

The mean age of patients in the JIA group was 12.4 years (SD: 3.5); in the non-JIA group, the mean age was 12.9 (SD:2.5), and the difference was not significant (*p* = 0.557).

Regarding sex of the patients, the JIA group consisted of 18 females (82%) and 4 males (18%), whereas in the non-JIA group there were 17 females (77%) and 5 males (23%), and the difference was not significant (*p* = 1).

The duration of arthritis in JIA patients was from 5 to 144 months (mean 35.41 months), and in non-JIA patients the duration of symptoms was from 6 to 36 months (mean 17.6 months).

Separation of the patient population into JIA and non-JIA was performed by pediatricians after the analysis of all available data—clinical, laboratory, and imaging. MRI was used to make a definitive diagnosis in many cases, in the light of non-conclusive clinical and blood tests.

On MRI only 6 children were lesions free, including 2 (4.5%) with JIA, and 4 (9.0%) in non-JIA group; *p* = 0.380. Inflammatory features were seen on MRI in 38 out of all the included patients (86%). Table 3 shows the frequency of MRI lesions in the JIA-confirmed vs. non-JIA groups in individual joints, tendon sheaths, bones, and entheses.

In general, more lesions were identified in the JIA group than in non-JIA.

The most common findings in both groups were effusion in the tibio-talar joint (68% in JIA, and 64% in the arthralgia group) and effusion in the subtalar joint (64% in JIA vs. 59% in the arthralgia non-JIA group). Tenosynovitis was the only lesion that was significantly more common in JIA vs. the non-JIA group (*p* = 0.031).

Regarding active inflammatory lesions, none of the non-JIA patients developed advanced active lesions (stages 2 or 3, depending on a lesion) and in none from the non-JIA group were the destructive lesions seen, whereas they were diagnosed in 12 out of 22 patients with JIA (55%). In none from the non-JIA group of patients the following lesions were seen: Kager’s fat pad inflammation, fat tissue inflammation in tarsal tunnel and in sinus tarsi, juxtaarticular soft tissue inflammation, or bursitis.

There was a very good interobserver agreement for scoring all active and destructive items, except for 2 cases of effusion in the inferior talar joint. The percentage of agreement was 96%, and Cohen’s kappa of agreement κ = 0.947 (95% CI: 0.875–1).

Since single MRI features were nonspecific for JIA (only the incidence of tenosynovitis was significantly higher in the JIA group), the MRI summarized score was calculated as the sum of the scores of an individual patient. The range of values of such an indicator ranged from 0 to 36 (Table 4). The more inflammatory features and the higher the score, the more likely the child has been diagnosed with JIA. However, even at a score of 16, a child was not diagnosed to have JIA, and specificity and PPV reached 100% with a score of 17 only.

The optimal cut-off point for the indicator appeared at the value of 10; at such a cut-off point, the best levels of sensitivity and specificity were obtained.

## 4. Discussion

This study showed that inflammatory features in the ankle and midfoot are frequently seen in MRI in children with a clinical diagnosis of arthritis, both with JIA as well without a final diagnosis of JIA. More lesions were identified in the JIA group than in non-JIA, but only tenosynovitis was significantly more common in the JIA vs. non-JIA group (*p* = 0.031). The MRI summarized score (a sum of all lesions’ scores), was unhelpful in discriminating ankle arthritis in JIA from non-JIA patients; the best levels of sensitivity (32%), specificity (91%), PPV (78%), and NPV (57%) were achieved only at a cut-off point of 10.

Talonavicular joint synovitis is particularly overlooked in clinical assessment [3]. Even limiting the clinical evaluation to soft tissues, it appears that differentiation between synovitis and tenosynovitis can be challenging on the basis of clinical evaluation, because these structures are in close proximity and both may cause diffuse swelling and decreased function [8,22,30,31]. One study reported tenosynovitis on MRI in more than half of the patients, whereas no tendon involvement had been detected clinically [8]. This was also confirmed by ultrasound studies [3,32,33] which found that clinical examination has low positive predictive value for synovitis and tenosynovitis assessment.

There have been a number of studies on hip, hand, and wrist, and especially knee MRI in JIA, but still very few have been undertaken on the ankle joint [3], although it is the second most frequently affected joint in children [3,4]. MRI provides a more objective, detailed, and reproducible assessment of disease status compared to clinical examination alone [8]. It can reliably evaluate the extent of inflammation in patients with JIA, including the detection of synovitis, tenosynovitis, and enthesitis [8,31,32], and it has the advantage over ultrasound in depicting BME and in showing areas of joint inflammation poorly visualized by ultrasound, such as sinus tarsi, tarsal canal, interosseous ligaments, or the posterior recess of the tibiotalar joint.

In this study we aimed to analyze in MRI the distribution and advancement of inflammatory lesions in the ankle joints and to test if MRI allows ankle arthritis in JIA to be differentiated from ankle arthralgia of unknown aetiology.

**Figure 1:**
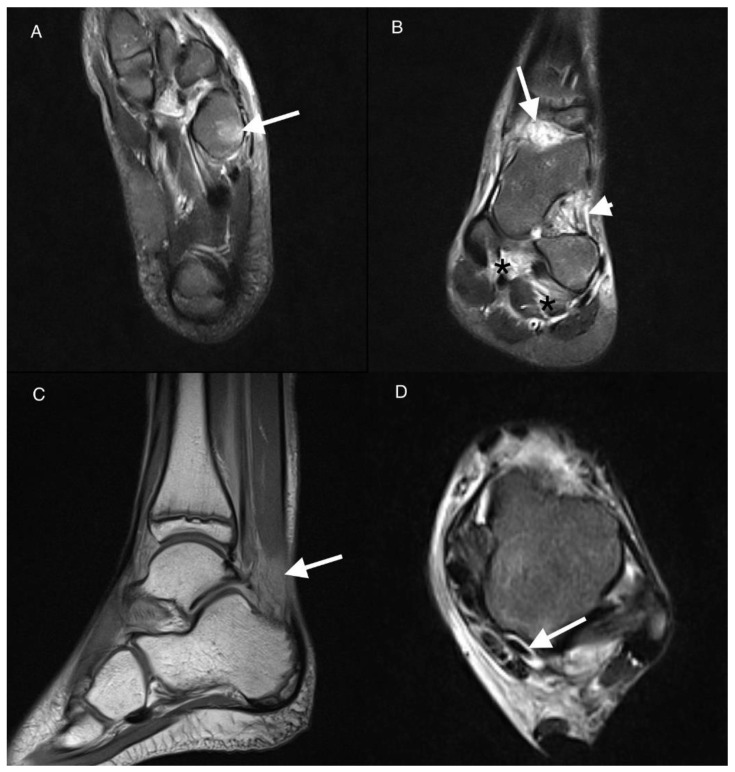
Magnetic resonance imaging (MRI) of the left ankle of an 11-year-old boy with juvenile idiopathic arthritis (JIA). T2-weighted turbo invertion recovery magnitude (TIRM) images in axial (**A**,**D**), coronal (**B**), and sagittal PD-weighted planes (**C**). Bone marrow edema (BME) stage 2 in the cuboid bone (arrow in (**A**)). Effusion/synovial thickening stage 2 in the tibio-talar joint (arrow in (**B**)). Fat tissue in sinus tarsi involvement stage 1 (arrowhead in (**B**)). Enthesitis of the interosseous ligaments stage 1 (black asterisks in (**B**)). Kager’s fat pad inflammation arrow in (**C**). Tenosynovitis of the flexor hallucis longus muscle tendon stage 1 arrow in (**D**).

According to the International League Against Arthritis (ILAR) classification of JIA, arthritis is the common feature of all JIA subtypes [3]. In this study arthritis joint effusion/synovial thickening was the most common lesion in both groups in the tibio-talar joint (68% in JIA vs. 64% in the arthralgia group) and in the subtalar joint (64% in JIA vs. 59% in arthralgia non-JIA group) (Figure 1). Surprisingly, the ILAR criteria do not include the involvement of other structures around the joint, such as tendon sheaths and entheses [3], or bone marrow or intraarticular or periarticular fat tissue. In this MRI evaluation, an exhaustive list of structures and tissues which potentially may be affected in the ankle joint were included.

In our study joint inflammation was more prevalent than tenosynovitis (69.7%) which is consistent with previous studies [8]. However, the study of Javadi et al. [8] included 46% patients with JIA receiving treatment before MRI, and 56% of the subjects received a gadolinium-based contrast agent. The tibiotalar joint was most frequently affected, like in the current study (Figure 1). Synovitis was also more prevalent in MRI than tenosynovitis in the Phatak et al. study [26] that enrolled 55 consecutive children with disease durations of less than 5 years. The tibio-talar joint was again the most frequently affected. The prevalence of subtalar joint involvement for Phatak et al. was similar to that of other studies [34].

**Figure 2:**
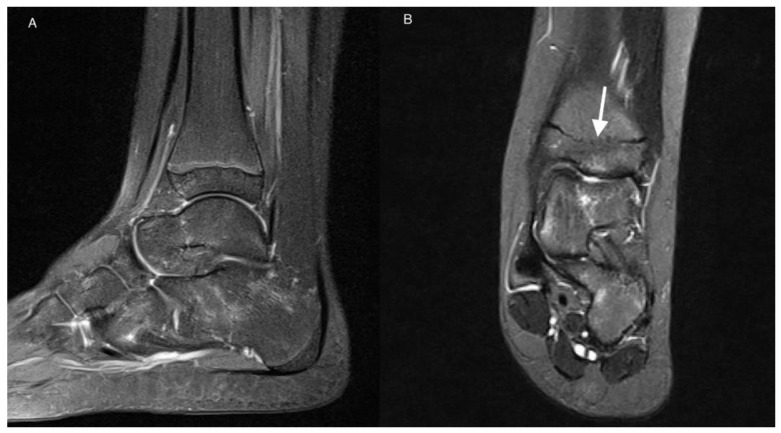
MRI of the left ankle of an 11-year-old girl with JIA. T2-weighted TIRM images in sagittal (**A**) and coronal (**B**) planes. Patchy BME (stage 1) in talus and calcaneus. Developmental disorder in the form of premature closure of physis (arrow).

Whereas BME was the most common pathology in a study by Phatak et al. [26] that focused on midfoot involvement only in ERA patients, in the current work, it was the second, after effusion/synovial thickening most commonly diagnosed lesion in both analyzed groups, even more frequently in the non-JIA group (Figure 2).

Tenosynovitis was seen less frequently than synovitis and BME in the current study—in 27%, all with JIA. However, it was the only feature which was significantly more common in the JIA vs. non-JIA group (*p* = 0.031). Long flexors and long extensors of the toes, flexor hallucis longus, and peroneus complex sheaths were affected, with two cases of secondary tendon involvement of the tibialis posterior tendon and flexor hallucis longus tendon. Tenosynovitis was detected by Javadi et al. [8] in 39% of patients, also less frequently than synovitis, and the tibialis posterior tendon (39%) and peroneus complex (18%) were the most commonly involved.

In other studies, tenosynovitis was more common than ankle joint arthritis [32,35]. It has been found in up to 71% of JIA patients with symptomatic ankle inflammation [35]. Isolated tenosynovitis (without synovitis) was reported from 3.9% of ankles to up to 39% of ankles [32]. Again, tibialis posterior tendon was the most commonly affected in MRI [8], followed by the peroneus longus and brevis tendons [4].

Enthesitis was seen on MRI in only 1 patient with the ERA subtype of JIA, with the involvement of the interosseous ligaments (Figure 1). Enthesitis is considered the leading feature of juvenile spondyloartropathies, pertaining to 3 subtypes of JIA according to ILAR classification: ERA, psoriatic arthritis, and undifferentiated arthritis. On clinical examination it was reported in even 80% of patients with ERA subtype, more commonly in juveniles than in adults [36], and involving mainly the hip, ankles, and feet [37]. Plantar fascia insertion (38%) and Achilles enthesis (22%) were one of most frequently affected sites in a cohort of patients with ERA [38]. US and MRI are important in the verification of both symptomatic and asymptomatic enthesitis which may have value in classification of children with JIA into these subtypes [26]. However, enthesopatic lesions can also be seen in 14% of healthy children [39].

Phatak et al. [26] conducted research focused on tarsitis that is believed to be characteristic of ERA subtype of JIA and is considered specific to juvenile SpA. The authors diagnosed inflammatory lesions in midfoot in 54% of included children in MRI, in comparison to 43% of the cases diagnosed on clinical examination and 36% seen on ultrasound. Enthesitis was seen in 25% of the cases mainly in tibialis posterior, flexor hallucis longus, peroneus longus, tibialis anterior, and peroneus brevis tendons. They also reported peroneus longus inflammation and adjacent bone marrow edema in cuboid in 8 patients which is called the functional enthesitis [12]. In the current study there was only one case of enthesitis of the midfoot interosseous ligaments.

Fat tissue involvement in ankle MRI in JIA has not been reported so far. In the current study MRI showed involvement of fat tissue in 3 patients with JIA: the Kager’s fat pad in 2 patients (10%), in sinus tarsi in 1 (5%), and in the tarsal canal in 1 (5%) (Figure 1). In RA fat tissue is infiltrated by the same inflammatory cells as synovium and subchondral bone and may be responsible for cartilage and bone damage [13]. Similar studies are missing for JIA.

**Figure 3:**
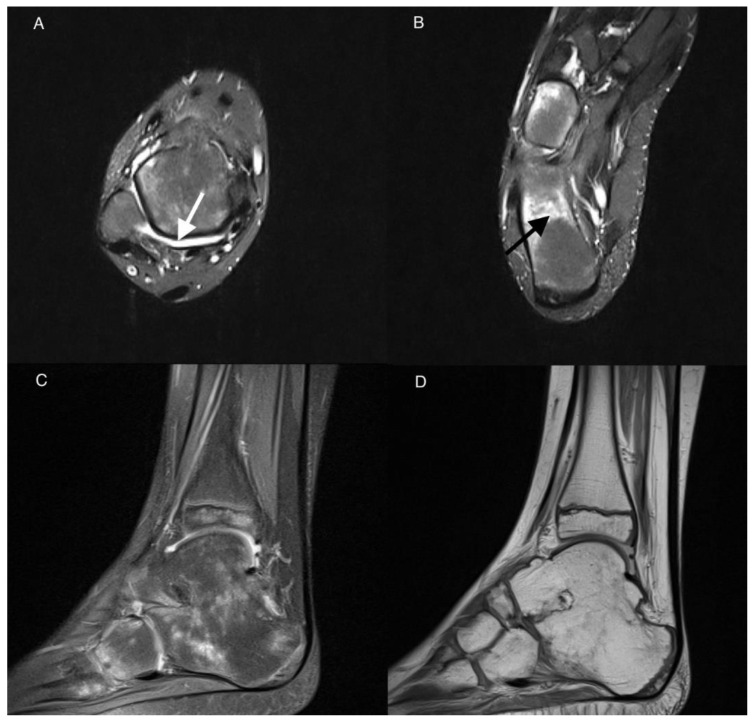
MRI of the right ankle of a 9-year-old boy with JIA. Axial T2-weighted TIRM images (**A**,**B**), sagittal PD with fat saturation (**C**), and sagittal PD-weighted image (**D**). Effusion/synovial thickening stage 2 in the tibio-talar joint (arrow). BME in numerous bones of the ankle and tarsum, the most advanced in calcaneus stage 3 (arrow in (**B**)). Tibio-talar joint space narrowing, tibio-calcaneal ankylosis, and osteophytes (**C**,**D**).

None of our 44 patients had myositis, a rare feature of JIA [40,41,42], or physis involvement. Destructive lesions were seen in single cases and in the JIA-confirmed group only (Table 3). Ankylosis, for example, was identified in only one patient in the talo-calcaneal joint (Figure 3). In a study by Phatak et al. [26], mentioned earlier, ankylosis tarsitis was diagnosed in 3 cases.

Developmental lesions were seen in both analyzed groups and were even more common in non-JIA (Table 4). In the JIA-confirmed group there were the Stieda process (1 patient) and premature closure of the distal physis of tibia and fibula (1 case) (Figure 2), and in non-JIA patients there were the Stieda process (2 cases), os trigonum (2 cases), and premature closure of the physis without features of past inflammation (1 case).

There was very high interobserver agreement for scoring all active and chronic items, except for 2 patients with effusion/synovial thickening in subtalar joint. Nevertheless, in the light of the propensity of the disease to involve the bone marrow, bone marrow lesions will especially require differentiation with edema-like signal changes representing residual hematopoietic marrow in the ankles and feet of healthy children. The latter are seen in up to 59% of patients younger than 16 years, are usually tiny, symmetrical, of fairly consistent pattern, are not associated with inflammatory features, and disappear with age [43,44]. However, the marrow pattern may be more extensive, confluent and may represent pathology; intensive focal or diffuse pattern of BME was observed in the JIA-only patients in the current study (Figure 1 and Figure 3).

In summary, this paper confirms that diagnosis of ankle arthritis in the course of JIA is challenging. Contrary to adults, where a majority of the ankle arthropathies result in osteoarthritis and trauma with quite specific clinical and radiographic presentations [45,46], in the pediatric population diagnosis is often based on exclusion criteria. Inflammatory features in MRI were seen with comparable frequency in children with JIA and without a final diagnosis of JIA, and out of a number of analyzed inflammatory and destructive features, only tenosynovitis was significantly more common in the JIA.

According to our knowledge, a similar large number of inflammatory lesions of the ankle joint has been lately proposed by Panwar and the MRI in JIA OMERACT working group [25] as a part of the standardized whole body-MRI scoring system aimed at assessing disease activity in juvenile idiopathic arthritis. Whereas our scoring system was used to test if MRI may help to differentiate ankle arthritis in the JIA from other arthropathies within the ankle and foot. The MRI summarized score was calculated as the sum of scorings of all 22 ankle and midfoot lesions in an individual patient to test which value provides the most optimal diagnosis in terms of high sensitivity, specificity, and positive and negative predictive values. The cut-off 10 points of the proposed MRI summarized score may support the differential diagnosis with high sensitivity and specificity for JIA.

A limitation of the paper was omitting the intravenous contrast administration in order to differentiate with higher accuracy joint and tendon sheath effusion from synovitis and tenosynovitis, but this was part of the protocol. Another issue that could influence the findings was preselection of the patients, who might have been first referred by clinicians for ultrasound and not diagnosed further, in case of positive ultrasound exam. That could result in reduction of the spectrum of lesions seen on MRI with predominance of BME over soft tissue abnormalities; the latter are well seen by ultrasound. Also, a selection bias might have resulted in the lack of structural damage on MRI in majority of this population, which is usually diagnosed on radiographs. On the other hand, damage rarely occurs early in JIA, and the low prevalence in the current study may mirror natural course of JIA.

The main advantage of the paper is involvement in the analysis of a number of possible MRI features that might be identified in JIA patients in ankle and midfoot. The MRI summarized scoring system aimed to discriminate JIA from non-JIA patients was also created and tested.

## 5. Conclusions

The findings of the study confirm that MRI diagnosis of JIA remains challenging, and except for tenosynovitis, other MRI features are nonspecific for JIA. The MRI summarized score, as a sum of all lesions’ scorings, also does not support discrimination between ankle arthritis in JIA from non-JIA patients with clinically suspected arthritis.

## Figures and Tables

**Table 1:** MRI acquisitions for ankle examination.

	Plane	TR	TE	ST (mm)	Gap (mm)	FoV (mm)	Matrix
Localiser	All	8.2	3.51	6.0	6.0	250 × 250	192 × 256
T2	Tra	7230	71	3.0	0.6	150 × 150	224 × 320
PD	Tra	2800	33	3.0	0.6	150 × 150	272 × 320
PD FS	Tra	2800	33	3.0	0.6	150 × 150	272 × 320
PD FS	Sag	2900	32	3.0	0.6	170 × 170	288 × 384
T1	Sag	666	11	3.0	0.6	170 × 170	240 × 320
T2 TIRM	Cor	4060	74	3.0	0.6	150 × 150	218 × 256
PD	Cor	3020	33	3.0	0.6	150 × 150	272 × 320

Cor, coronal; FOV, field of view; FS, fat saturation; Sag, sagittal; ST, slice thickness; TE, echo time; TR, repetition time; Tra-transverse; w, weighted.

**Table 2:** MRI scoring system for ankle joint arthritis.

	Inflammatory Features	Scoring
1	Effusion/synovial thickening	0: no intraarticular fluid 1: trace of fluid not distending the joint capsule/physiologic 2: mild: increased amount of fluid/synovial thickening mildly distending joint capsule 3: moderate to severe: increased amount of fluid/synovial thickening moderately to severely distending joint capsule
2	Bone marrow edema * ^#^	0: no BME 1: discrete patchy BME 2: focal BME 3: diffuse BME
3	Tenosynovitis	0: no tenosynovitis 1: tenosynovitis 2: tenosynovitis with secondary tendinitis
4	Enthesitis of the tendons, plantar fascia, ligaments	0: no enthesitis 1: enthesitis—at least one of the enthesis’ inflammatory features is present: high signal and/or thickening of the enthesis and/or perientheseal soft tissue inflammation and/or BME in the bony part of the enthesis
5	Bursitis	0–1
6	Myositis	0–1
7	Juxtaarticular soft tissue inflammation	0–1
8	Kager’s fat pad involvement	0–1
9	Fat tissue in tarsal tunnel involvement	0–1
10	Fat tissue in sinus tarsi involvement	0–1
11	Bone erosions	0–1
12	Cysts	0–1
13	Chondromalacia	0–1
14	Joint space narrowing	0–1
15	Physis involvement	0–1
16	Ankylosis	0–1
17	Osteophytes	0–1
18	Sclerotization	0–1
19	Avascular necrosis	0–1
22	Developmental disorders	0–1

* Bone area pertaining to Achilles and plantar fascia attachment belongs to the enthesitis domain; BME in physis pertain to physis involvement domain. ^#^ In case of different stages of BME seen in a given bone, the highest score is applied.

**Table 3:** MRI results in JIA vs. non-JIA group.

	MRI Lesions and Scorings	JIA Confirmed Group *n* = 22	Non-JIA Group *n* = 22	*p*
1	Effusion/Synovial thickening tibio-talar joint			
	0	7 (32%)	8 (36%)	0.352
	1	9 (41%)	11 (50%)	
	2	3 (14%)	3 (14%)	
	3	3 (14%)	0 (0%)	
2	Effusion/Synovial thickening subtalar joint			
	0	8 (36%)	9 (41%)	0.490
	1	7 (32%)	9 (41%)	
	2	5 (23%)	4 (18%)	
	3	2 (9%)	0 (0%)	
3	BME tibia			
	0	16 (73%)	19 (86%)	0.415
	1	5 (23%)	3 (14%)	
	2	1 (5%)	0 (0%)	
	3	0 (0%)	0 (0%)	
4	BME fibula			
	0	17 (77%)	20 (91%)	0.385
	1	4 (18%)	2 (9%)	
	2	1 (5%)	0 (0%)	
	3	0 (0%)	0 (0%)	
5	BME calcaneus/subtalar joint			
	0	15 (68%)	17 (77%)	0.547
	1	5 (23%)	5 (23%)	
	2	1 (5%)	0 (0%)	
	3	1 (5%)	0 (0%)	
6	BME talus			
	0	15 (68%)	18 (82%)	0.433
	1	6 (27%)	4 (18%)	
	2	1 (5%)	0 (0%)	
	3	0 (0%)	0 (0%)	
7	BME naviculare			
	0	16 (73%)	18 (82%)	0.347
	1	4 (18%)	4 (18%)	
	2	2 (9%)	0 (0%)	
	3	0 (0%)	0 (0%)	
8	BME cuboideum			
	0	18 (82%)	17 (77%)	0.191
	1	2 (9%)	5 (23%)	
	2	2 (9%)	0 (0%)	
	3	0 (0%)	0 (0%)	
9	BME cuneiform medial			
	0	18 (82%)	18 (82%)	0.565
	1	3 (14%)	4 (18%)	
	2	1 (5%)	0 (0%)	
	3	0 (0%)	0 (0%)	
10	BME cuneiform intermedium			
	0	18 (82%)	18 (82%)	0.565
	1	3 (14%)	4 (18%)	
	2	1 (5%)	0 (0%)	
	3	0 (0%)	0 (0%)	
11	BME cuneiform lateral			
	0	20 (91%)	18 (82%)	0.234
	1	1 (5%)	4 (18%)	
	2	1 (5%)	0 (0%)	
	3	0 (0%)	0 (0%)	
12	BME base of MET1			
	0	20 (91%)	20 (91%)	0.513
	1	1 (5%)	2 (9%)	
	2	1 (5%)	0 (0%)	
	3	0 (0%)	0 (0%)	
13	BME MET2			
	0	21 (95%)	21 (95%)	0.368
	1	0 (0%)	1 (5%)	
	2	1 (5%)	0 (0%)	
	3	0 (0%)	0 (0%)	
14	BME MET3			
	0	21 (95%)	21 (95%)	0.368
	1	0 (0%)	1 (5%)	
	2	1 (5%)	0 (0%)	
	3	0 (0%)	0 (0%)	
15	BME MET4			
	0	21 (95%)	21 (95%)	0.368
	1	0 (0%)	1 (5%)	
	2	1 (5%)	0 (0%)	
	3	0 (0%)	0 (0%)	
16	BME MET5			
	0	21 (95%)	21 (95%)	0.368
	1	0 (0%)	1 (5%)	
	2	1 (5%)	0 (0%)	
	3	0 (0%)	0 (0%)	
17	Enthesitis			
	0	21 (95%)	22 (100%)	0.660
	1	1 (5%)	0	
18	Tenosynovitis			
	0	16 (73%)	22 (100%)	0.031
	1	4 (18%)	0 (0%)	
	2	2 (9%)	0 (0%)	
19	Kager’s fat pad inflammations			
	0	20 (91%)	22 (100%)	1
	1	2 (10%)	0 (0%)	
20	Fat tissue inflammation in tarsal tunnel			
	0	21 (95%)	22 (100%)	1
	1	1 (5%)	0 (0%)	
21	Fat tissue inflammation in sinus tarsi			
	0	21 (95%)	22 (100%)	1
	1	1 (5%)	0 (0%)	
22	Juxtaarticular soft tissue inflammation			
	0	21 (95%)	22 (100%)	1
	1	1 (5%)	0 (0%)	
24	Bursitis			
	0	20 (91%)	22 (100%)	0.469
	1	2 (9%)	0 (0%)	
25	Myositis			
	0	22 (100%)	22 (100%)	1
	1	0 (0%)	0 (0%)	
26	Cyst			
	0	21 (95%)	22 (100%)	1
	1	1 (5%)	0 (0%)	
27	Bone erosion			
	0	20 (91%)	22 (100%)	0.469
	1	2 (9%)	0 (0%)	
28	Chondromalacia			
	0	20 (91%)	22 (100%)	0.469
	1	2 (9%)	0 (0%)	
29	Joints space narrowing			
	0	20 (91%)	22 (100%)	0.469
	1	2 (9%)	0 (0%)	
30	Physis involvement			
	0	22 (100%)	22 (100%)	1
	1	0 (0%)	0 (0%)	
31	Ankylosis			
	0	21 (95%)	22 (100%)	1
	1	1 (5%)	0 (0%)	
32	Osteophytes			
	0	20 (91%)	22 (100%)	0.469
	1	2 (9%)	0 (0%)	
33	Sclerotization			
	0	21 (95%)	22 (100%)	1
	1	1 (5%)	0 (0%)	
34	AVN/OCD			
	0	21 (95%)	22 (100%)	1
	1	1 (5%)	0 (0%)	
35	Developmental lesions			
	0	20 (91%)	17 (77%)	0.410
	1	2 (9%)	5 (23%)	

**Table 4:** Diagnostic value of the summarized MRI score as a predictor of JIA.

MRI Score	JIA	Non JIA	True Pos.	False Pos.	False Neg.	True Neg.	Sensitivity	Specificity	PPV	NPV
36	1	0	1	0	21	22	0.045	1.000	1.000	0.512
21	1	0	2	0	20	22	0.091	1.000	1.000	0.524
17	1	0	3	0	19	22	0.136	1.000	1.000	0.537
16	0	1	3	1	19	21	0.136	0.955	0.750	0.525
14	1	0	4	1	18	21	0.182	0.955	0.800	0.538
13	0	1	4	2	18	20	0.182	0.909	0.667	0.526
11	2	0	6	2	16	20	0.273	0.909	0.750	0.556
10	1	0	7	2	15	20	0.318	0.909	0.778	0.571
9	0	1	7	3	15	19	0.318	0.864	0.700	0.559
7	0	1	7	4	15	18	0.318	0.818	0.636	0.545
6	0	1	7	5	15	17	0.318	0.773	0.583	0.531
4	2	3	9	8	13	14	0.409	0.636	0.529	0.519
3	1	1	10	9	12	13	0.455	0.591	0.526	0.520
2	6	5	16	14	6	8	0.727	0.364	0.533	0.571
1	4	4	20	18	2	4	0.909	0.182	0.526	0.667
0	2	4	22	22	0	0	1.000	0.000	0.500	

Pos.—positive, neg.—negative.
